# Descending thoracic aortic aneurysm revealing metastasis of a soft tissue fibrosarcoma: a case report and review of the literature

**DOI:** 10.1186/s13569-018-0109-7

**Published:** 2018-10-26

**Authors:** Clotilde Delerce, Olivia Bailly, Amine Bouhamama, Sophie Couchon, Frank Pilleul, Arnaud Thivolet, Charles Mastier

**Affiliations:** 1 Department of Interventional Radiology, Centre Léon Bérard, 28 rue Laennec, 69008 Lyon, France; 2 Department of Medical Oncology, Centre Léon Bérard, 28 rue Laennec, 69008 Lyon, France; 3 CREATIS, UMR CNRS 5220-INSERM 1206, Centre LCC, 28 rue Laennec, Lyon, France

**Keywords:** Soft tissue sarcoma, Aortic aneurysm, Aortic metastasis

## Abstract

**Background:**

Review of the first documented case of aortic wall metastasis from a limb sarcoma.

**Case presentation:**

In a 56-year-old woman with a diagnosis of a high-grade limb fibrosarcoma, an aortic metastasis was revealed by a fast growing aneurysm of the descending thoracic aorta. This was managed with an endoprosthesis.

**Conclusion:**

The presence of an aneurysm in a patient with a sarcoma with a high potential for metastasis and poor cardiovascular risk factors should alert physicians.

## Background

Soft-tissue sarcoma is a rare entity (it represents less than 1% of malignant tumors [1]). These tumors have a propensity for locally destructive growth and recurrence, and 20% to almost 100% of them have a significant risk of distant metastasis, depending on their histological type and grade. One-tenth of soft-tissue sarcomas are already in a metastatic state when discovered [2]. Due to the poor prognosis in the case of metastatic neoplasia [3], a baseline CT scan is recommended for tumor staging, especially for sarcomas measuring more than five centimeters. Recent studies also suggest that positron emission tomography (PET) may be clinically indicated to evaluate biological activity in soft tissue masses [4], but this technique can lead to false positives due to its low specificity in the case of infectious or active inflammatory processes. There are no clear current guidelines regarding management of metastatic sarcoma [5].

Typically, metastatic sarcomas are considered incurable and palliative treatment, including chemotherapy, is performed (for performance status = 0–2). Tumors spread primarily through the vascular system, and lungs are the most common site for distant metastasis. To our knowledge, an aortic metastasis of a sarcoma has not yet been described as primary intimal sarcomas are most commonly reported in the literature [6]. In this report, we describe a case of aortic metastasis of a limb fibrosarcoma revealed by a descending thoracic aortic aneurysm, and its management.

## Case report

**Fig. 1:**
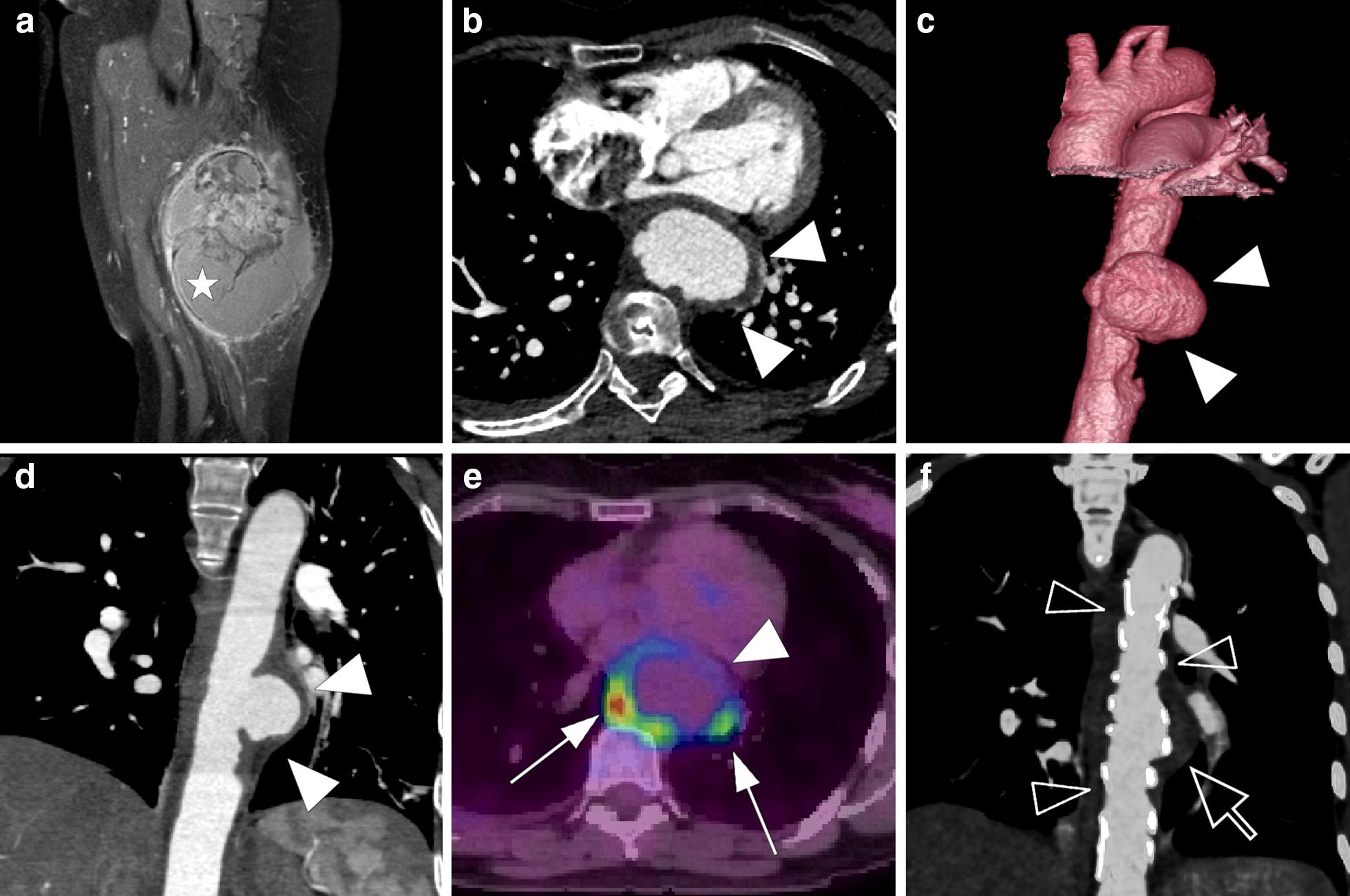
**a** Coronal gadolinium-enhanced T1-weighted image with fat saturation shows a posterolateral muscular mass of the left thigh with both necrotic and enhanced portions (white star); Saccular aneurysm of the descendant thoracic aorta (white head arrows): arterial phase CT scan of the aneurysm with axial view (**b**), coronal reconstruction (**c**) and 3D reconstruction (**d**). **e** PET-CT image fusion shows FDG uptake (white arrows) around the aneurysm. **f** Coronal maximum intensity projection (MIP) reconstruction of postoperative CT shows the endoprosthesis (transparent head arrows) and the excluded aneurysmal cavity (transparent arrow)

A 56-year-old nonsmoking woman consulted for a painful mass in her left thigh. Her past medical history was only significant for untreated autoimmune hepatitis. MRI of her left thigh showed a posterolateral muscular mass measuring five centimeters, with both necrotic and enhanced portions (Fig. 1a). Histologic analysis performed on biopsy samples showed evidence of malignant pleomorphic proliferation suggesting a diagnosis of high-grade fibrosarcoma. However, the results were not totally conclusive because of pan-cytokeratin AE1/AE3 expression that could also be found in sarcomatoid carcinomas. The diagnosis of pleomorphic fibrosarcoma was finally maintained due to the lack of epithelial marker CK7, CK5/6 and p63. (French Federation of Cancer Centers Sarcoma Group grading system [FNCLCC] = Differentiation: 3; Necrosis: 2; mitosis: 10 High-Power Field: 35; Mitotic Index: 3; Grade: 3).

A thoracoabdominal CT scan was performed for tumor staging and found a 35-mm saccular aneurysm of the descending thoracic aorta (Fig. 1b–d). The patient, despite her age, had no cardiovascular risk factors. A PET-CT performed 1 month later showed a suspicious contralateral limb metastasis and abnormal aortic FDG uptake around the aneurysm, which could be attributed to an infectious or tumorous process (Fig. 1e). Follow-up CT scans showed quick growth of the aneurysm from 35 to 49 mm. The patient was then transferred for endovascular aortic repair with an endoprosthesis (Fig. 1f) measuring 28 × 164 mm (Relay NBS^®^ Bolton Medical). The presence of an atypical epigastric artery angiogram led to an artery biopsy during the procedure, which found no evidence of dysplasia. Despite negative blood samples taken near the aneurysm, the possibility of an infectious location was discussed due to the patient’s poor dental condition. Postoperative probabilistic antibiotic treatment was started with oxacillin and ofloxacin, and then amoxicillin.

Surgical resection of the primary tumor was rejected due to histological confirmation of a right gluteal metastasis (contralateral). Initially, the patient received conventional chemotherapy by doxorubicin and ifosfamide, but the treatment was quickly switched to cisplatin and paclitaxel due to tumor progression. The patient was then included in a clinical trial (NCT01308034 Study of Continuous Dosing of Sunitinib in Non GIST Sarcomas with Concomitant Radiotherapy) and treatment by sunitinib was introduced with concomitant radiotherapy. Due to adverse effects that led to a deep thrombocytopenia (50 G/L), sunitinib was stopped.

**Fig. 2:**
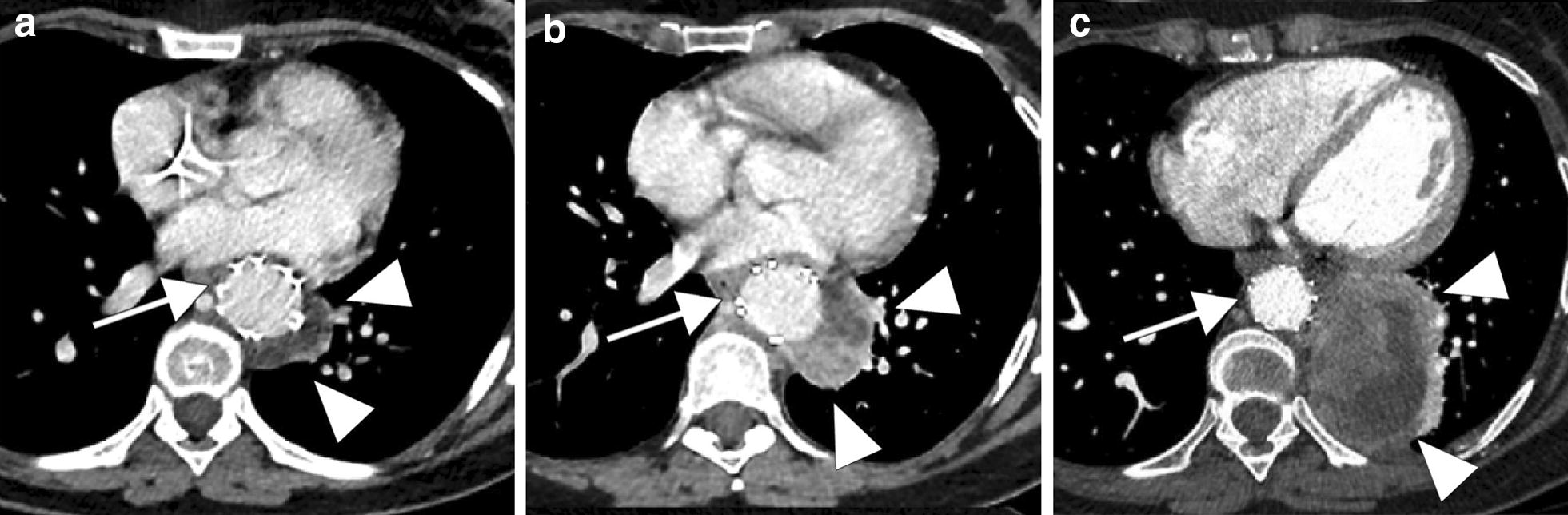
Follow-up CT scans shows rapid progression of the lateral aortic metastasis (white head arrows) near the endoprosthesis (white arrows). **a** Initial postoperative CT scan shows a non-enhancing lateral aortic lesion which is difficult to differentiate from an excluded aneurysmal cavity. **b** One month later CT scan shows an important tumor’s size increase with a minimal peripheral enhancement (**b**). **c** Six months later, CT scan shows an important tumor progression

**Fig. 3:**
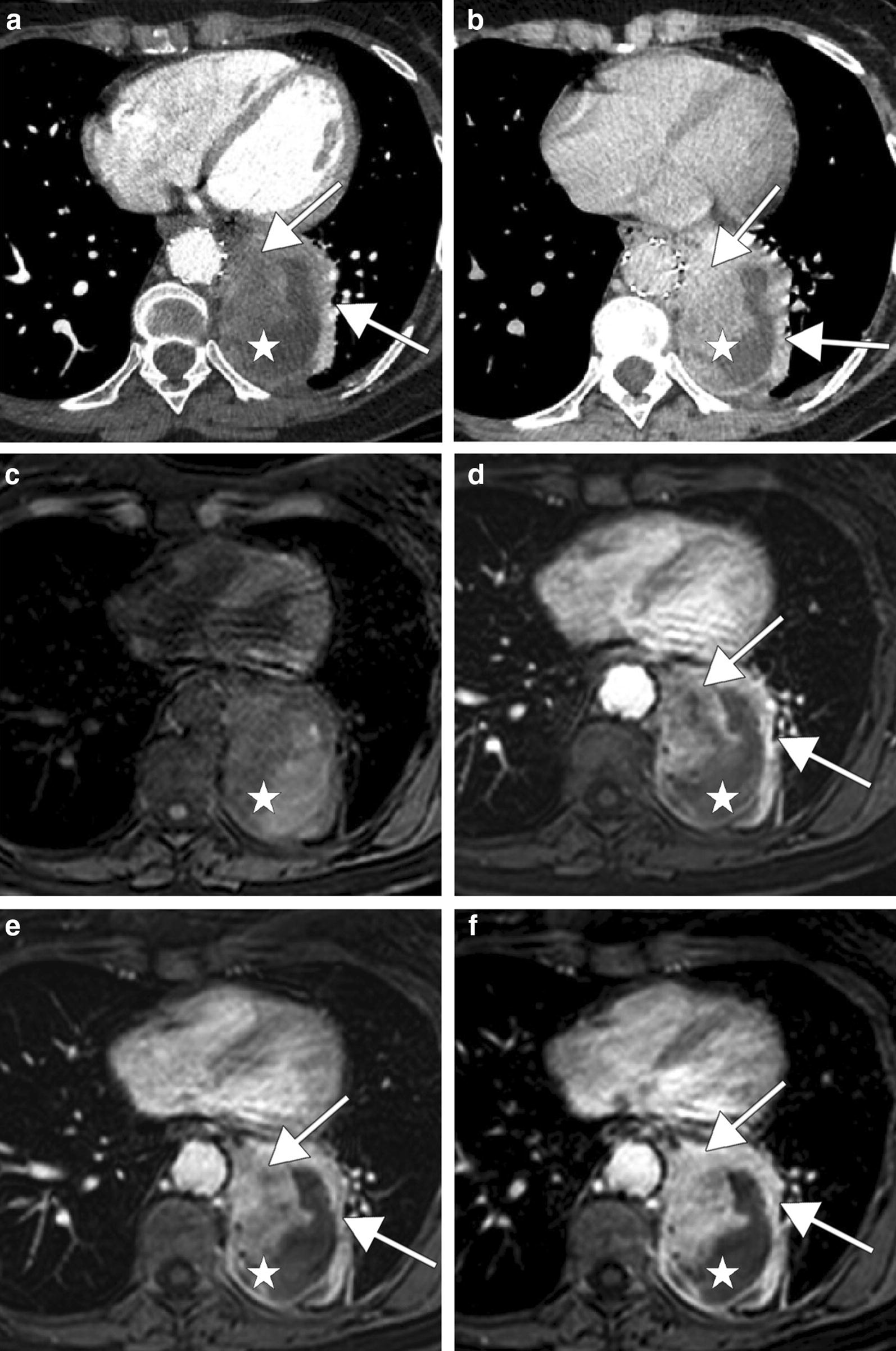
CT scan and MRI performed 6 months after endoprosthesis placement show a large lateral aortic tumor mass (white stars) with a progressive contrast enhancement (white arrows). CT scan shows a minimal contrast enhancement on portal phase (**a**) and an important one on delayed phase (**b**). Axial non-contrast (**c**) and gadolinium-enhanced T1-weighted images with fat saturation on arterial phase (**d**), portal phase (**e**) and delayed phase (**f**) show also a progressive tumor enhancement

Follow up CT scans (Fig. 2a–c) showed the appearance of a suspicious lesion near the excluded aneurysmal cavity with contrast-enhanced portions (Fig. 3a, b). Aortic MRI and contrast-enhanced ultrasound confirmed the presence of tumor tissue instead of thrombotic material within the aortic aneurysm sac (Fig. 3c–f). A transparietal biopsy of the large mass was performed using ultrasound guidance by a left posterior paravertebral approach, and histological examination found pleomorphic spindle cells with pan cytokeratin and smooth muscle actin positivity which confirmed the diagnosis of sarcoma metastasis (FNCLCC grade 3). Palliative management was decided. The patient was included in another clinical trial (NCT02406781-PEMBROSARC) and received four injections of pembrolizumab during a 3-month period, associated with cyclophosphamide. The patient died due to mediastinal tumor progression 17 months after initial sarcoma diagnosis.

## Discussion

The luminal-type intimal sarcoma (IS) is the most common sarcoma of the aorta [7]. In the early stages, tumor aneurysms are very similar to atherosclerotic aneurysms on CT findings, which often leads to a delay in diagnosis. Tumor etiology must be considered in the case of low cardiovascular risk factors, atypical imaging findings or a rapidly evolving aneurysm. In the late stages, multiplanar imaging often helps to discriminate tumor tissues from atherosclerotic materials and shows enhancing lesions around the aneurysm and tumor extending into the aortic wall or adjacent structures.

Sarcoma survival rates are closely linked to their potential metastasis risk. Since 2002, the World Health Organization (WHO) Classification of Tumors of Soft Tissue and Bone divided these tumors into three risk groups: benign, intermediate (locally aggressive or rarely metastasizing*)* and malignant. This classification helps physicians adapt treatment according to the risk of tumor metastasis. There are no current guidelines regarding initial tumor staging, but the WHO clearly recommends multidisciplinary decision-making. Sarcomas spread primarily through the vascular system, and the lungs are the most common site for distant metastasis. Patients at high risk of metastasis usually have an initial chest CT scan; a thoracoabdominal CT scan may be also performed.

Sarcoma rarely metastasizes to the skin, soft tissues, liver or lymph nodes (3%). Primary aortic sarcomas are rare tumors, with approximately 30 cases reported in the literature [8].

Typically, patients with aortic intimal sarcomas have symptoms caused by tumor emboli including pulseless extremities or abdominal pain, rather than symptoms directly related to the primary tumor [9]. In our case, the patient was asymptomatic and there was no evidence of aortic lumen obstruction.

[18F]-2-Fluorodeoxyglucose (FDG) is the most commonly used tracer for PET imaging. This is a glucose analog that accumulates in cells and FDG uptake reflects tissue metabolic activity. In oncology, it is used to detect metastases by virtue of their metabolic differences compared to surrounding normal tissue. False positives commonly occur with active inflammatory or infectious lesions and false negatives occur with low metabolic activity tumors or small lesions (< 1 cm), due to limited resolution. In our case, PET-CT was used in conjunction with initial CT scan as a complementary diagnostic tool and showed intense FDG uptake around the aneurysm. However, in the case of rapidly evolving atherosclerotic aneurysms, FDG uptake can also be seen. An aortic metastatic localization was not initially considered to be the most likely diagnosis in our case. Follow-up CT scans and MRI findings found enhanced tissues around the aneurysm.

The endovascular treatment of aortic aneurysms is now the gold standard to avoid the risk of rupture beyond 5 cm. In this case, simple monitoring was impossible in view of rapid progression, and the multidisciplinary team judged open surgery to be inappropriate and too invasive.

To our knowledge, an aortic metastasis of a sarcoma has not yet been reported in the literature. Immunohistochemical tests are needed to discriminate primary intimal sarcomas from other aortic tumors. Indeed, intimal sarcomas do not express epithelial, smooth muscle, or schwannoma-like markers and are positive for mesenchymal marker, vimentin. In our case, pan cytokeratin and smooth muscle actin markers were positive, similar to the primitive limb sarcoma.

## Conclusion

Aortic metastases of limb sarcoma are rare. In the early stages, tumor aneurysms are very similar to atherosclerotic aneurysms on CT findings, which often lead to a delay in diagnosis. The atypical localization of the aneurysm, low cardiovascular risk factors, rapidly evolving aneurysm and enhanced tissues around the aorta are suspicious findings for aortic metastasis. FDG uptake around the aneurysm, although not specific, can contribute to the diagnosis.
